# Systematic expression analysis of ligand-receptor pairs reveals important cell-to-cell interactions inside glioma

**DOI:** 10.1186/s12964-019-0363-1

**Published:** 2019-05-22

**Authors:** Dongsheng Yuan, Yiran Tao, Geng Chen, Tieliu Shi

**Affiliations:** 1 Center for Bioinformatics and Computational Biology, and Shanghai Key Laboratory of Regulatory Biology, Institute of Biomedical Sciences, School of Life Sciences, East China Normal University, Shanghai, 200241 China; 2 National Center for International Research of Biological Targeting Diagnosis and Therapy, Guangxi Key Laboratory of Biological Targeting Diagnosis and Therapy Research, Collaborative Innovation Center for Targeting Tumor Diagnosis and Therapy, Guangxi Medical University, Nanning, China

**Keywords:** Glioma, Single-cell RNA-seq, Cell-to-cell interactions, Machine learning

## Abstract

**Background:**

Glioma is the most commonly diagnosed malignant and aggressive brain cancer in adults. Traditional researches mainly explored the expression profile of glioma at cell-population level, but ignored the heterogeneity and interactions of among glioma cells.

**Methods:**

Here, we firstly analyzed the single-cell RNA-seq (scRNA-seq) data of 6341 glioma cells using manifold learning and identified neoplastic and healthy cells infiltrating in tumor microenvironment. We systematically revealed cell-to-cell interactions inside gliomas based on corresponding scRNA-seq and TCGA RNA-seq data.

**Results:**

A total of 16 significantly correlated autocrine ligand-receptor signal pairs inside neoplastic cells were identified based on the scRNA-seq and TCGA data of glioma. Furthermore, we explored the intercellular communications between cancer stem-like cells (CSCs) and macrophages, and identified 66 ligand-receptor pairs, some of which could significantly affect prognostic outcomes. An efficient machine learning model was constructed to accurately predict the prognosis of glioma patients based on the ligand-receptor interactions.

**Conclusion:**

Collectively, our study not only reveals functionally important cell-to-cell interactions inside glioma, but also detects potentially prognostic markers for predicting the survival of glioma patients.

**Electronic supplementary material:**

The online version of this article (10.1186/s12964-019-0363-1) contains supplementary material, which is available to authorized users.

## Background

Glioma is the most common primary central nervous system (CNS) tumor in adults, and is known for its high heterogeneity and poor clinical outcomes [1]. Traditional researches regarding the expression profile of glioma were mainly based on bulk RNA-seq technologies, which mainly provided us an initial view of gene expression at cell-population level. Tumors are usually comprised of heterogeneous cells that differ in many biological features, such as morphology, proliferation, invasion, metastasis and drug resistance [2]. Thus, bulk RNA-seq data reflects the averaged expression profile of potentially different cells, which fails to reveal the intrinsic expression differences among distinct cell subpopulations, leading to the ignorance of cell heterogeneity [3]. Single-cell RNA-sequencing (scRNA-seq) technologies enable us to gain insight into the transcriptome at single-cell resolution and allow us to have a deeper understanding of intra-tumor heterogeneity. The advancements of scRNA-seq largely facilitated the development of novel approaches to improve targeted therapy and precision medicine [4, 5].

Tumor together with surrounding stromal cells and extracellular matrix (ECM) constitute a tumorous niche referred as the tumor microenvironment (TME) [2], which plays crucial roles in each step of tumorigenesis [6]. In gliomas, TME is comprised of immune cells, astrocytes, oligodendrocytes and neurons, while most of immune cells are macrophages and microglia (> 95%) [7, 8]. Macrophages have been reported to be preferentially enriched in the tumor core region, but how tumor associated macrophages (TAMs) in TME affect the tumor biological process has not been fully understood. Venteicher et al. revealed that IDH-A gliomas were highly infiltrated by microglia/macrophage cells, but they did not explore the interactions between tumor cells and macrophages in gliomas [9]. Cancer stem cells (CSCs) is also a small subpopulation of tumor cells with the ability of self-renew, differentiate and responsible for drug resistance and cancer recurrence [10–12]. CSCs and TAMs are enriched around blood vessels [13, 14], and both of them are important for promoting tumor growth by intercellular signaling to support diverse biological processes [15, 16]; however, the interactions between TAMS and CSCs are less explored. Cell-to-cell communications between diverse cell types are mediated by specific pairs of secreted ligands and cell-surface receptors. Chakrabarti et al. found that macrophages may nourish stem cells and the stem cells could secrete ligand DLL1 to activate Notch pathway to increase the expression level of Wnt ligand in macrophages for promoting the function and survival of stem cells [17]. CSCs of gliomas were also found to recruit TAMs by secreting POSTN to support the growth of glioblastoma (GBM) [18]. Nevertheless, those findings are done by functional experiments, which are time-consuming and limited to a single interaction each time. The scRNA-seq data provide great opportunities for interrogating the genome-wide crosstalk between glioma CSCs and TAMs.

Here, we first explored the cell types of glioma cells using manifold learning based on a large amount of scRNA-seq data. Then the autocrine interactions among neoplastic cells were analyzed. Furthermore, we investigated the gene expression profile of macrophages and CSCs in gliomas, and subsequently examined the crosstalk between the two kinds of cell types. In the end, we built a robust machine learning model to predict the survival risk of glioma patients based on the expression level of specific ligands and receptors.

## Methods

### Gene expression data

We downloaded the single-cell RNA-seq gene expression data of glioma from GEO (Gene Expression Omnibus, https://www.ncbi.nlm.nih.gov/geo/) with accession number GSE89567. Bulk RNA-seq data of glioma were downloaded from The Cancer Genome Atlas (TCGA) data portal (https://tcga-data.nci.nih.gov/). Our analysis mainly focused on low grade glioma (LGG) and glioblastoma (GBM).

### Validation dataset

We used the RNA-seq data of a cohort of 325 glioma patients from Chinese Glioma Genome Atlas (CGGA, http://www.cgga.org.cn/) [19] as validation dataset for the machine learning model.

### Ligand-receptor pairs database

The ligand-receptor pairs analyzed in this study were from public dataset provided by a previous research [20].

### Statistical analysis

Our analyses were performed with R software, version 3.4.4 (http://www.R-project.org). We performed preliminary clustering of all cells using t-SNE based on single-cell RNA-seq gene expression data by employing monocle2 (https://github.com/cole-trapnell-lab/monocle-release) [21]. Differential gene expression calling was conducted by MAST (https://github.com/RGLab/MAST) [22]. Spearman’s correlation coefficients were calculated by the function of ‘cor’ in R basic package (version 3.4.4).

### Machine learning model

Extreme Gradient Boosting (XGBoost) [23] was used to train the model to predict clinical outcomes. We used Python 2.7 anaconda 3–4.0.0 to build the machine learning model. Python package sklearn [24] was applied in machine learning training. We randomly split the TCGA gliomas dataset into training and test sets with 3:1 ratio. XGBoost is the most advanced classifier based on decision trees. More details about XGBoost algorithm can be found in the explanatory document written by the algorithm designer (https://xgboost.readthedocs.io/en/latest/).

## Results

### Neoplastic and healthy cell clusters are identified in glioma

**Fig. 1:**
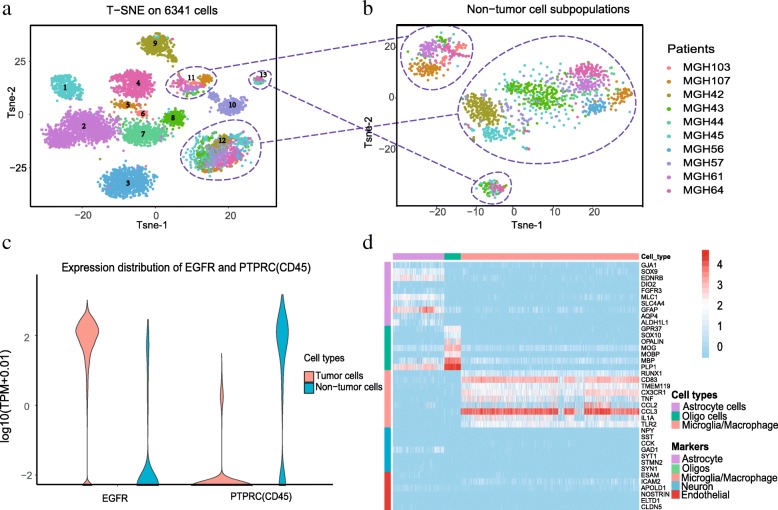
Neoplastic and non-neoplastic clusters in gliomas. **a** T-distributed stochastic neighbor (t-SNE) embedding plot of 6341 cells of 10 glioma patients. **b** T-SNE plot of non-neoplastic cells from gliomas. **c** Violin plot of expression of EGFR and PTPRC (CD45) in neoplastic and non-neoplastic cells. **d** Expression heatmap of some known markers in non-neoplastic cells. Note: the t-SNE maps of (**a**) and (**b**) were conducted based on the top 5% highly variable genes in expression

To gain insights into the subpopulations and cellular diversity of gliomas, we first analyzed the scRNA-seq data of 6341 IDH-A glioma cells from 10 patients [9] by performing t-distributed stochastic neighbor embedding (t-SNE) based on the top 5% highly variable genes in expression. As shown in Fig. 1a, clusters 1 to 10 were separately composed of the cells from a single one of 10 patients, whereas clusters of 11–13 were mixed with the cells originating from distinct patients (Fig. 1b). Since tumor cells are more heterogeneous than normal cells, the cells in clusters 1–10 could be neoplastic, while the cells in clusters 11–13 might be healthy cells. We further validated the neoplastic and healthy cell clusters based on the expression profile of the known markers of mature human neurons astrocytes, oligodendrocytes, microglia/macrophages, and endothelial cells [25]. Previous study showed that the expression of neoplastic cell markers like EGFR can be used to identify neoplastic cells with high sensitivity and specificity [7]. We also found that neoplastic cell clusters of gliomas express significantly higher level of EGFR than that of healthy cell clusters (Fig. 1c). However, those neoplastic cell clusters barely expressed the myeloid-specific gene PTPRC (CD45), but this gene was broadly expressed in healthy cells.

To further explore the TME of non-neoplastic cells in glioma, we used the reported marker genes for different residential cell types to define the identity of each cell cluster [25]. As expected, we observed large expression differences among several cell types including neurons, oligodendrocytes, microglia/macrophages, and endothelial cells, in those three healthy cell clusters (Fig. 1d). We then annotated the cells with known marker genes and found that microglia/macrophages occupy the largest portion of healthy cells (Fig. 1d).

### Macrophage and microglia cells are distinguished from non-neoplastic clusters

**Fig. 2:**
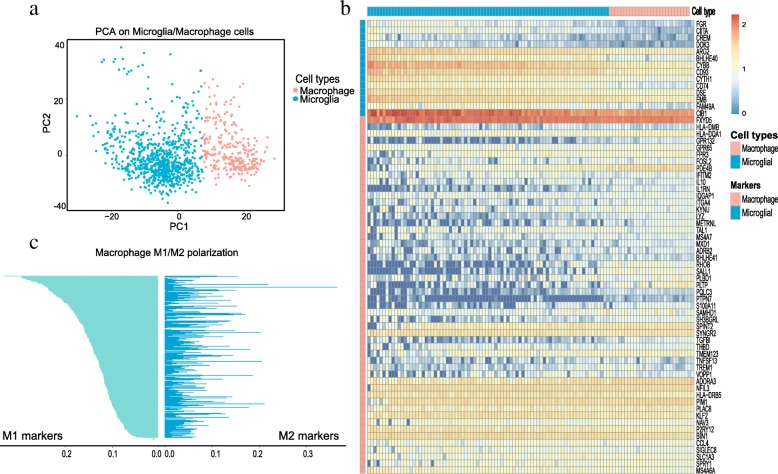
Distinguishing macrophage and microglia by gene signatures. **a** PCA of macrophage and microglia cells based on signature genes. The 237 human-mouse homologous genes reported previously were used to plot PCA. **b** Heatmap of macrophage and microglia cells based on 66 maker genes. **c** Distribution of M1/M2 scores (average expression of M1/M2 marker genes) for macrophages. Rows are ranked by M1 scores

Previous study suggested that 237 human-mouse homologs are enough to discriminate discrete human glioma TAMs from macrophage/microglia cluster through the principal component analysis (PCA) based on scRNA-seq data [26]. We then explored those 237 genes in the macrophage/microglia cell cluster of glioma using PCA and found that macrophage and microglia cells are stratified into two subpopulations, respectively (Fig. 2a). Moreover, Muller et al. previously identified 66 potential maker genes that were strongly differently expressed between macrophage and microglia cells [26]. We also observed that those 66 marker genes of macrophage/microglia are respectively enriched in the two subpopulations identified by us, which further confirmed that one subpopulation is macrophages while the other is microglia (Fig. 2b).

To investigate the polarization heterogeneity of macrophages, we compared the expression level of specific markers for M1 (e.g. CD64, SOCS1 IDO, CD86, CD80, IL1R1, TLR2 and TLR4) and M2 (such as MRC1, TGM2, CCL22, CD163, TLR1, TLR8 and CLL22) macrophages [27]. Intriguingly, we found that the markers of M1/M2 co-exist in macrophages and no significant correlation is observed between the marker expression of M1 and M2 (Fig. 2c). Accordingly, our results further confirm previous finding that macrophages may have mixed M1/M2 phenotypes.

### Expression correlation analysis reveals significant autocrine ligand-receptor pairs in glioma

**Fig. 3:**
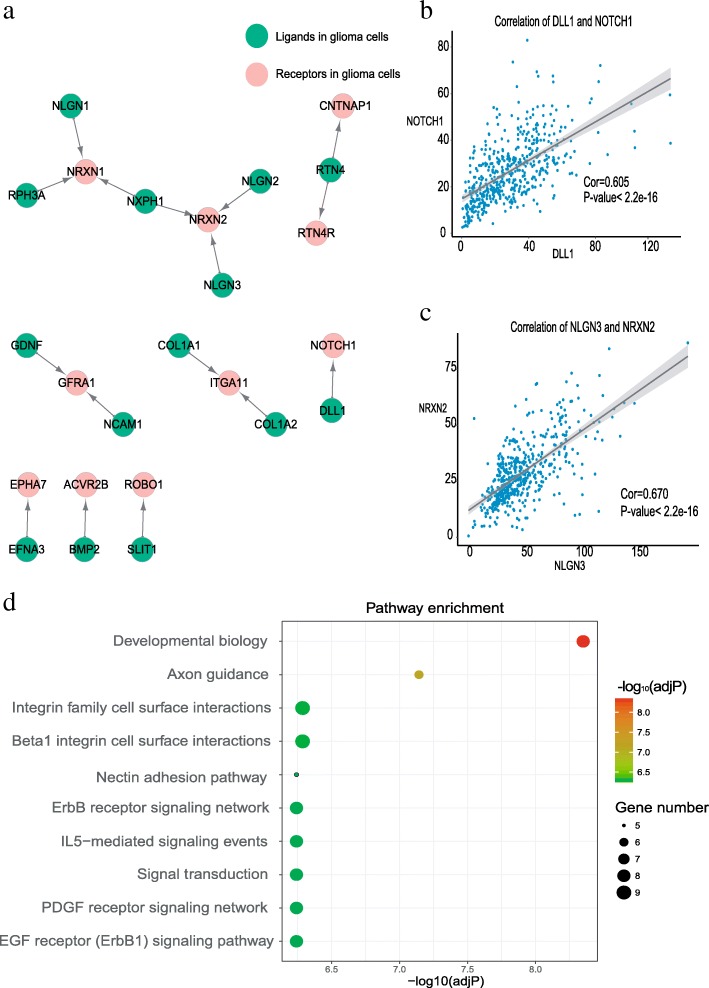
The autocrine ligand-receptor signaling network identified in glioma cells. **a** Ligand-receptor pairs of autocrine signals inside glioma neoplastic cells. Dots stand for ligands and receptors and arrows points from ligands to receptors. A total of 16 pairs with significant Spearman’s correlations were identified (*P*-value <0.05). **b** (**c**) Spearman’s correlation coefficients of two ligand-receptor pairs (DLL1-NOTCH1 and NLGN3-NRXN2) in TCGA LGG dataset. **d** Significantly enriched pathways for autocrine ligand-receptor pairs in tumor cells (adjusted *P*-value <0.05)

Since the expression level of ligands and their receptors could reflect the level of cell-to-cell communication [28], we tried to identify the autocrine network among glioma neoplastic cells. We first collected 696 ligands and 653 cognate receptors (2419 pairs of chemokine/cytokine) from a public interaction dataset [20], and then compared their expression between neoplastic and non-neoplastic cells. By screening the expression of both receptors and ligands increased in glioma neoplastic cells with MAST [22], 186 ligand-receptor pairs were detected. Considering that co-expression of a ligand and its corresponding receptors is necessary for cell-cell communication through secreting signals, thus we subsequently calculated the Spearman’s correlation coefficients for the 186 ligand-receptor pairs using the TCGA Low-Grade Glioma (LGG) dataset [29]. A total of 16 pairs with significant Spearman’s correlation coefficients higher than 0.4 (*P*-value < 0.05) were identified (Fig. 3a, Additional file 1: Table S1). Furthermore, down-regulation of NOTCH1 and its ligand DLL1 has been reported to inhibit tumor proliferation in glioma cell lines, which may play the function of promoting tumor proliferation [30]. Our finding showed that NOTCH1 and DLL1 are both highly expressed in tumor cells (Fig. 3b). Additionally, previous research suggested that the secretion of NLGN3 could promote glioma growth [31], and we found that its binding partners are also highly expressed in tumor cells (Fig. 3c).

Specifically, GDNF (Glial Cell Derived Neurotrophic Factor) has been hypothesized to promote tumor growth and invasion in prostate cancer and its effect of resisting treatment could be related to the expression of its receptor GFRA1 [32]. We found that GDNF together with its two receptors in autocrine ligand-receptor pairs are highly expressed in glioma neoplastic cells (Additional file 1: Figure S1a). We also conducted gene functional enrichment analysis and found that those 16 ligand-receptor pairs are mainly involved in the pathways of tumor invasion, cell adhesion and cytoskeleton, cell growth and proliferation (Fig. 3d). Therefore, those 16 ligand-receptor pairs identified by us have important roles and may be tightly associated with the development of glioma.

### The crosstalk between CSCs and macrophages is associated with the survival of glioma patients

To discover the crosstalk between CSCs and macrophages based on ligands and receptors, we first selected the stem-like cells with high expression of the 90 genes associated with stemness in glioma (Additional file 1: Table S2) [9]. Then we compared the expression of those ligands and receptors between stem-like and other differentiated tumor cells. The ligands and receptors that were with higher expression in macrophages than that of microglia were further identified. Next, we explored the cell-to-cell communications between stem-like cells and macrophages based on those highly expressed ligand-receptor pairs.

**Fig. 4:**
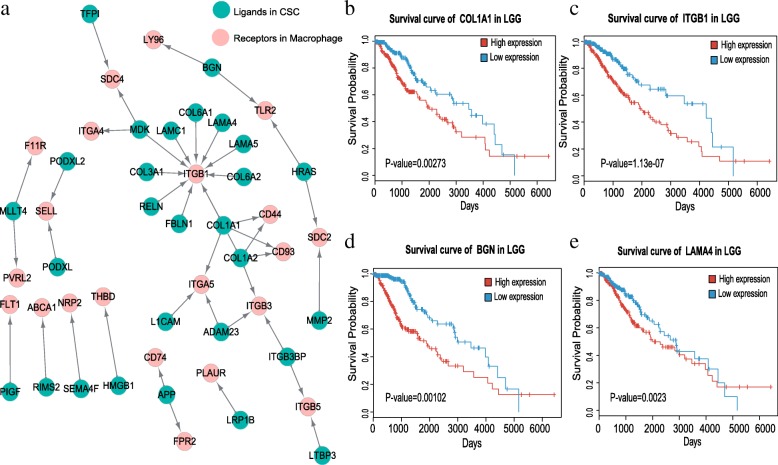
The crosstalk from CSCs to macrophages. **a** Ligand-receptor pairs of the signaling network from CSCs to macrophages. Green dots stand for ligands highly expressed in CSCs and red dots represent highly expressed in receptors in macrophages (adjusted *P*-value <0.05). The arrows point from ligands to receptors. In total, 42 ligand-receptor pairs were identified. **b**-**e** Kaplan-Meier survival analysis for COL1A1, ITGB1, BGN and LAMA4 in TCGA LGG dataset (*n* = 512 and *P*-value <0.05)

Interestingly, the crosstalk between stem-like cells and macrophages that showed ligands highly expressed in stem-like cells and receptors highly expressed in macrophages was mainly related to invasion and angiogenesis (Fig. 4a, Additional file 1: Figure S2). We found that the CSCs highly express some ligands encoding collagens I and III, such as COL1A1, COL1A2, COL3A1, COL6A1 and COL6A2. A previous study suggested that cancer cells and leukocytes could migrate instantly along collagen fibers [33]. Our results also implied similar mechanism that those ligand-receptor pairs may help glioma cells to migrate in glioma. Moreover, the receptors that highly expressed in macrophages, like CD44, ITGB1, ITGB3 and ITGA5, contained the integrin members. Integrins are known to directly bind the components of extracellular matrix (ECM) and could provide the traction necessary for cell motility and invasion [34, 35]. Intriguingly, we observed that the patients with high expression of COL1A1, ITGB1 and ITGB3 are prone to with significantly poor prognostic outcomes in TCGA LGG datasets (Fig. 4b c, f and Additional file 1: Figure S3; *P*-value < 0.05). Furthermore, our result shows that BGN (Biglycan) is highly expressed in CSCs, and BGN has been reported to promote tumor invasiveness via inducing integrin-β1 expression [36]. Additionally, laminins (e.g. LAMA4, LAMA5 and LAMC1) may take part in the interactions between CSCs and macrophages as well. We found that the glioma patients with higher expression of LAMA4 is significantly associated with shorter survival days (Fig. 4e, *P*-value <0.05).

Besides, we also identified a ligand-receptor pair that related to angiogenesis. Previous research suggested that PLGF (Placenta growth factor) could recruit macrophage lineage cells in inflammatory sites [37] [38]. We observed that CSCs remarkably express PLGF and its only receptor VEGFR1 is highly expressed in macrophages which may help macrophages enrich in tumor site. Thus our result implies that CSCs could recruit macrophages via PLGF-VEGFR1 system.

### The ligands in macrophages and the receptors in CSCs are also correlated with patient survival

**Fig. 5:**
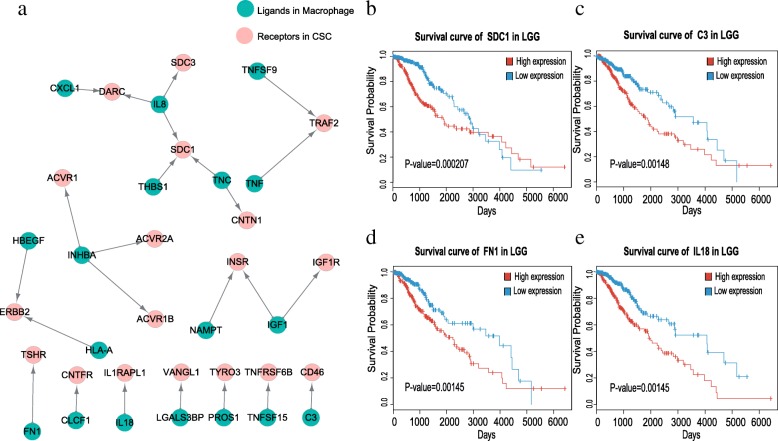
The crosstalk from macrophages to CSCs. **a** Ligand-receptor pairs of the signaling network from macrophages to CSCs. Green dots stand for ligands highly expressed in macrophages and red dots represent receptors highly expressed in CSCs. The arrows point from ligands to receptors (adjusted P-value <0.05.). The crosstalk contains a total of 24 significant ligand-receptor pairs. **b**-**e** Kaplan-Meier survival analysis for SDC1, C3, FN1 and IL18 in TCGA LGG dataset (*n* = 510 and P-value <0.05)

To further explore the interactions between CSCs and macrophages, we analyzed how macrophages communicated with CSCs via ligand-receptor pairs. For the ligands and receptors separately highly expressed in macrophages and CSCs, we identified 24 ligand-receptor pairs that showed crosstalk from macrophages to stem-like cells (Fig. 5a). We found that these 24 ligand-receptor pairs are mainly involved in the pathways of angiogenesis, integrin and apoptosis (Additional file 1: Figure S4).

It has been suggested that TNF expressed by macrophages can promote angiogenesis through IL-8 and VEGF to help tumor cells escape from immune system [39] [40]. Moreover, IL-8 has been shown to be strongly correlated with brain cancer grades [41, 42]. However, we found that IL-8 is mainly expressed by macrophages rather than tumor cells in glioma. Furthermore, we observed that the patients with lower expression of SDC1 is significantly associated with longer survival days (Fig. 5b, *P*-value <0.05). The high expression of SDC1 is crucial for promoting stem-like cell invasion and metastasis in cell-extracellular matrix adhesion [43]. C3 plays a key role in complement system and could support tumor growth by immunosuppression as well [44]. We observed that C3 is highly expressed in macrophages, and the glioma patients with higher expression of C3 tend to have significantly decreased survival days (Fig. 5c, *P*-value <0.05). Other ligand-receptor pairs many also influence tumor progress in different ways. For example, Kast et al. suggested that FN1 may play a key role in extracellular matrix components to increase tumor invasion and IL-18 could promote tumor growth [45]. Our result indicates that both FN1 and IL-18 are significantly associated with poor prognosis of glioma patients (Fig. 5d and e, *P*-value <0.05). In total, 34 ligand and receptor genes in the crosstalk between CSCs and macrophages are significantly associated with survival in the LGG dataset (*P*-value <0.05).

### Prognostic model based on ligand-receptor interactions achieves high accuracy

To predict the prognosis of glioma patients, we built a model based on the ligand-receptor interactions between macrophages and CSCs using advanced machine learning algorithm XGBoost [46]. Firstly, we performed principle component analysis (PCA) based on the genes of ligand-receptor pairs in IDH-mutated and IDH-wildtype gliomas, and found that IDH-mutated and IDH-wildtype gliomas mixed together in PCA (Additional file 1: Figure S5), suggesting that these two types of gliomas have little difference in the expression of the ligand-receptor pairs. Then, we randomly chose 75% of the gene expression dataset of all the TCGA glioma samples as training set and the rest of 25% as test set. Then the training set was used to train a XGBoost model to predict the prognosis of glioma patients. We observed that this XGBoost model achieve a precision of 0.80 and a recall of 0.78 in the test set, which is much better than models built on randomly selected genes (Additional file 1: Figure S6).

**Fig. 6:**
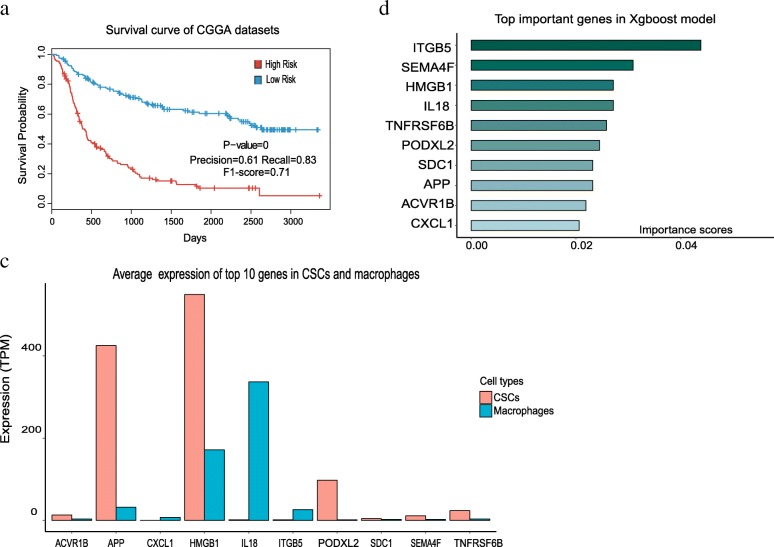
Prognostic predictor for glioma patients based on XGBoost. **a** The performance of the prognostic predictor Kaplan-Meier survival analysis for the patients in CGGA dataset (*n* = 315 and P-value =0). **b** Importance rank of the top 10 genes in the prognostic classifier. Importance scores stand for the importance of genes in the predicting model. **c** Bar chart of average expression for the top 10 genes in CSCs and macrophages. TPM: transcript per million

To further validate the result of our XGBoost model, we tested the RNA-seq data of 325 glioma samples from CGGA (Chinese Glioma Genome Atlas) [19]. Strikingly, our model can significantly divide the patients into high-risk and low-risk groups as well, and the survival time of these two patient groups are remarkably different (*P*-value = 0), which further confirmed the performance of our robust model (Fig. 6a). The expression level of ITGB5 is the most important feature in the model followed by SEMA4F, IL18 and HMGB1 (Fig. 6b). Among the top 10 important genes, ITGB5, IL18 and CXCL1 are highly expressed in macrophages, while the receptors of SCD1, TNFRSF6B, ACVR1B are highly expressed in CSCs and the ligands of PODXL2, HMGB1, SEMA4F and APP are mainly expressed in CSCs (Fig. 6c). In addition, previous studies suggested that ITGB5 and IL18 are related to tumor invasiveness [40, 43], whereas CXCL1 and SEMA4F are associated with cell proliferation [47, 48]. Moreover, HMGB1 and SCD1 have been demonstrated to be closely correlated with drug resistance in glioma [49, 50]. However, little is known about the role of ACVR1B and PODXL2 on gliomas.

## Discussion

The evolution of tumor is a complicate progress, which involves the interactions between neoplastic and non-neoplastic cells infiltrating in the tumor microenvironment. The advantages of scRNA-seq provide unprecedented opportunities to investigate the cell-to-cell communications inside tumor. In this study, we systematically explored the communication networks between different cell types in glioma and gained insights into tumor microenvironment.

In the microenvironment of gliomas, different kinds of cells were infiltrating and macrophages were enriched in tumor core site. We revealed that tumor cells could accomplish some biological processes through autocrine and paracrine ways. For instance, the neurite outgrowth inhibitor (Nogo) of RTN4 could play a newfound role in carcinogenesis through AKT signaling pathway, and knockdown of RTN4 could postpone tumor proliferation in mice [51]. We found that RTN4 and its receptors are highly expressed in the neoplastic cells of glioma (Additional file 1: Figure S1B), indicating that glioma cells may promote tumor proliferation through autocrine RTN4 signaling. Besides, glioma cells could also induce macrophages via producing diverse ligands and intriguing macrophages in tumor niche. In return, macrophages in tumor site stimulate tumor processes and facilitate tumor progression [39]. We identified possible interactions in gliomas based on large-scale scRNA-seq data and some of those interactions have been proved to play important roles in tumor development. Moreover, our results were further validated using the TCGA bulk RNA-seq data of glioma. The ligand-receptor pairs identified by us could provide potential guidance and reference for experimental design.

In the crosstalk inside gliomas, the ligand-receptor signal pairs were associated with various biological processes, some of which may influence tumor progression. For instance, IL-8 has been shown to be strongly correlated with brain cancer grades [41, 42], and it was barely detected in normal CNS area but highly expressed in brain cancers [39]. We found that IL-8 is mainly expressed by macrophages in TME rather than glioma cells, and its receptor, SDC1, is highly expressed in CSCs. BGN were highly expressed in CSCs and its cell surface receptors of TLR2 and LY96, encoding toll-like receptors 2 and 4, were highly expressed in macrophages. High content of reactive oxygen species (ROS) accumulated in hypoxia environment could promote tumor proliferation and the inhibition of toll-like receptors 2 and 4 may restrain ROS functions [52]. Therefore, BGN secreted by CSCs may function with toll-like receptors 2 and 4 in macrophages to help CSCs to survive in hypoxia and maintain rapid growth. In addition, CD46 (membrane cofactor protein), could facilitate the inactivation of C3b by serum factor I [53], but the role of CD46 in protection tumor cells from the attack of complement system remains unknown. We observed that CD46 is highly expressed in CSCs which may defense CSCs from the attack of complement-mediated inflammatory response. This may explain that CSCs are able to survive in the environment with high content of C3 secreted by macrophages and escape the attack from complement system.

Since the ligands and receptors identified by us were significantly associated with the clinical outcomes of glioma, we built a robust model to predict the survival risk of patients to help clinical decisions using the advanced machine learning algorithm. Although the outcomes of patients could relate to the factors of age and general health conditions, our model still achieved good performance. Accordingly, our identified possible interactions in gliomas could be useful for the treatment and drug design of glioma [54, 55].

Taken together, our results provide a landscape of the autocrine interactions in glioma neoplastic cells as well as the crosstalk between macrophages and glioma stem-like cells, which may facilitate the identification of potential therapeutic targets for precision medicine.

## Conclusions

Glioma is known for its heterogeneity and worse prognostic outcomes. In this study, we firstly revealed the cell-to-cell interactions inside gliomas using manifold learning based on a large amount of scRNA-seq data. Further analyses of the scRNA-seq and TCGA RNA-seq data of glioma provide a landscape of the autocrine interactions in glioma neoplastic cells as well as the crosstalk between macrophages and glioma stem-like cells. Some ligands and receptors identified by us could significantly affect prognostic outcomes and function via different pathways. Our machine learning model is able to accurately predict the prognosis of glioma patients based on the ligand-receptor interactions.

## Additional file

Additional file 1: **Table S1.** 16 autocrine ligand-receptor pairs with significant Spearman’s correlation coefficients higher than 0.4. the first time they are cited. **Table S2.** 90 genes associated with stemness in glioma. **Figure S1.** Spearman’s correlation coefficients of two ligand-receptor pairs (GDFR-GFRA1 and RTN4-CNTNAP1) in TGCA LGG dataset. **Figure S2.** Enriched pathways for ligands highly expressed in stem-like cells and receptors highly expressed in macrophages (Pathway commons). **Figure S3.** Kaplan-Meier survival analysis for ITGB3 in TCGA LGG dataset. **Figure S4.** Enriched pathways for ligands highly expressed in macrophages and receptors highly expressed in stem-like cells (Pathway commons). **Figure S5.** PCA of IDH-mutated and IDH-wildtype gliomas based on the genes of ligand-receptor pairs. **Figure S6.** Performance of model built by randomly selected genes. (DOCX 546 kb)

## Abbreviations

BGN: Biglycan
CGGA: Chinese Glioma Genome Atlas
CNS: central nervous system
CSCs: cancer stem-like cells
ECM: extracellular matrix
GBM: glioblastoma
GDNF: Glial Cell Derived Neurotrophic Factor
LGG: low grade glioma
PCA: Principal components analysis
ROS: reactive oxygen species
scRNA-seq: single-cell RNA sequencing
TAMs: tumor associated macrophages
TCGA: The Cancer Genome Atlas
TME: tumor microenvironment
t-SNE: t-Distributed Stochastic Neighbor Embedding
