# Supplementary material for: Systematic expression analysis of ligand-receptor pairs reveals important cell-to-cell interactions inside glioma
Source: Cell Commun Signal. 2019 May 22;17:48. doi: 10.1186/s12964-019-0363-1 (PMC6532229; doi:10.1186/s12964-019-0363-1)
Supplement: Supplementary file 1 — Table S1. 16 autocrine ligand-receptor pairs with significant Spearman’s correlation coefficients higher than 0.4. the first time they are cited. Table S2. 90 genes associated with stemness in glioma. Figure S1. Spearman’s correlation coefficients of two ligand-receptor pairs (GDFR-GFRA1 and RTN4-CNTNAP1) in TGCA LGG dataset. Figure S2. Enriched pathways for ligands highly expressed in stem-like cells and receptors highly expressed in macrophages (Pathway commons). Figure S3. Kaplan-Meier survival analysis for ITGB3 in TCGA LGG dataset. Figure S4. Enriched pathways for ligands highly expressed in macrophages and receptors highly expressed in stem-like cells (Pathway commons). Figure S5. PCA of IDH-mutated and IDH-wildtype gliomas based on the genes of ligand-receptor pairs. Figure S6. Performance of model built by randomly selected genes. (DOCX 546 kb) [file 12964_2019_363_MOESM1_ESM.docx]

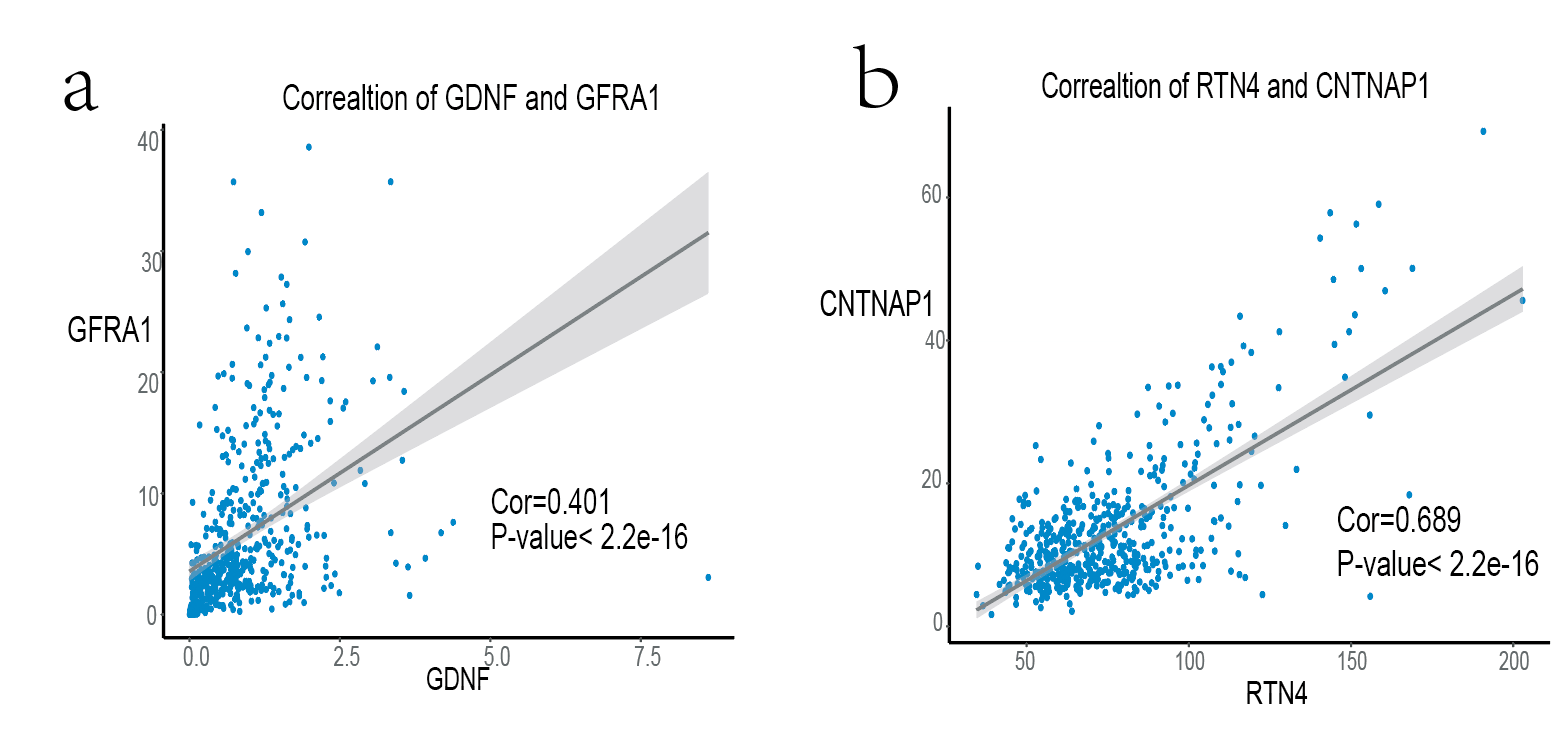


**Supplementary Figure 1**. Spearman’s correlation coefficients of two ligand-receptor pairs (GDFR-GFRA1 and RTN4-CNTNAP1) in TGCA LGG dataset.


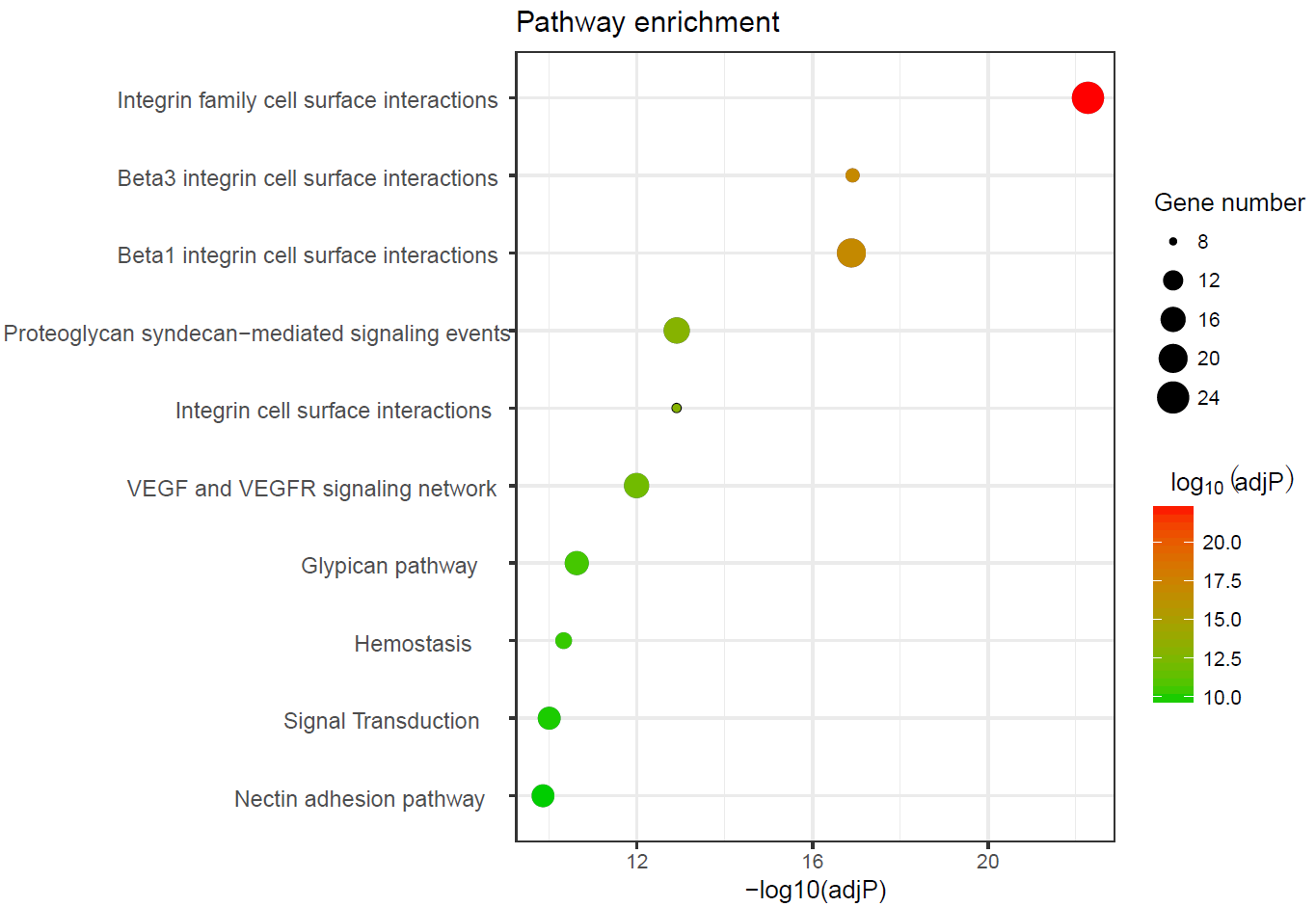


**Supplementary Figure 2**. Enriched pathways for ligands highly expressed in stem-like cells and receptors highly expressed in macrophages (Pathway commons).


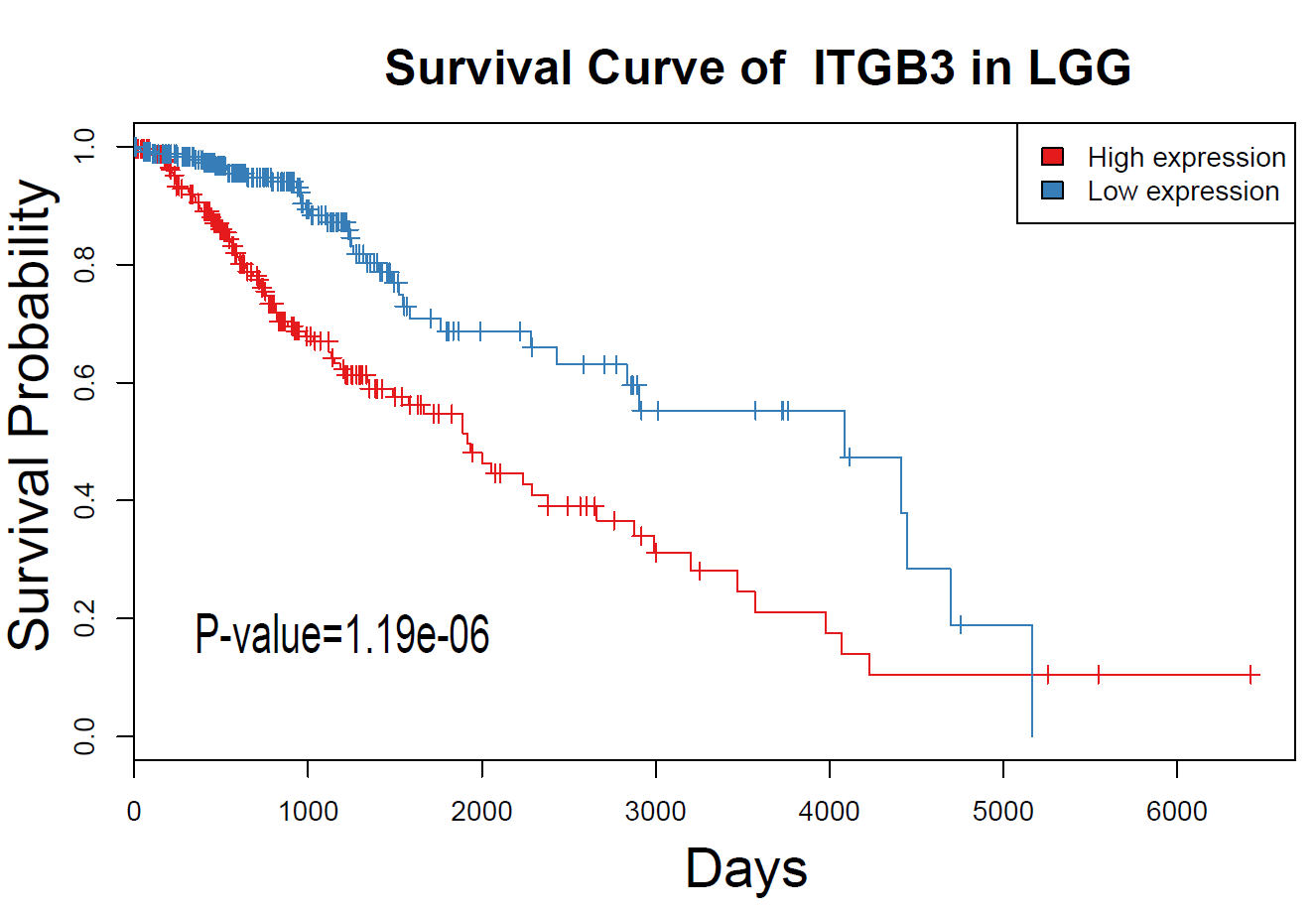


**Supplementary Figure 3.** Kaplan-Meier survival analysis for ITGB3 in TCGA LGG dataset.


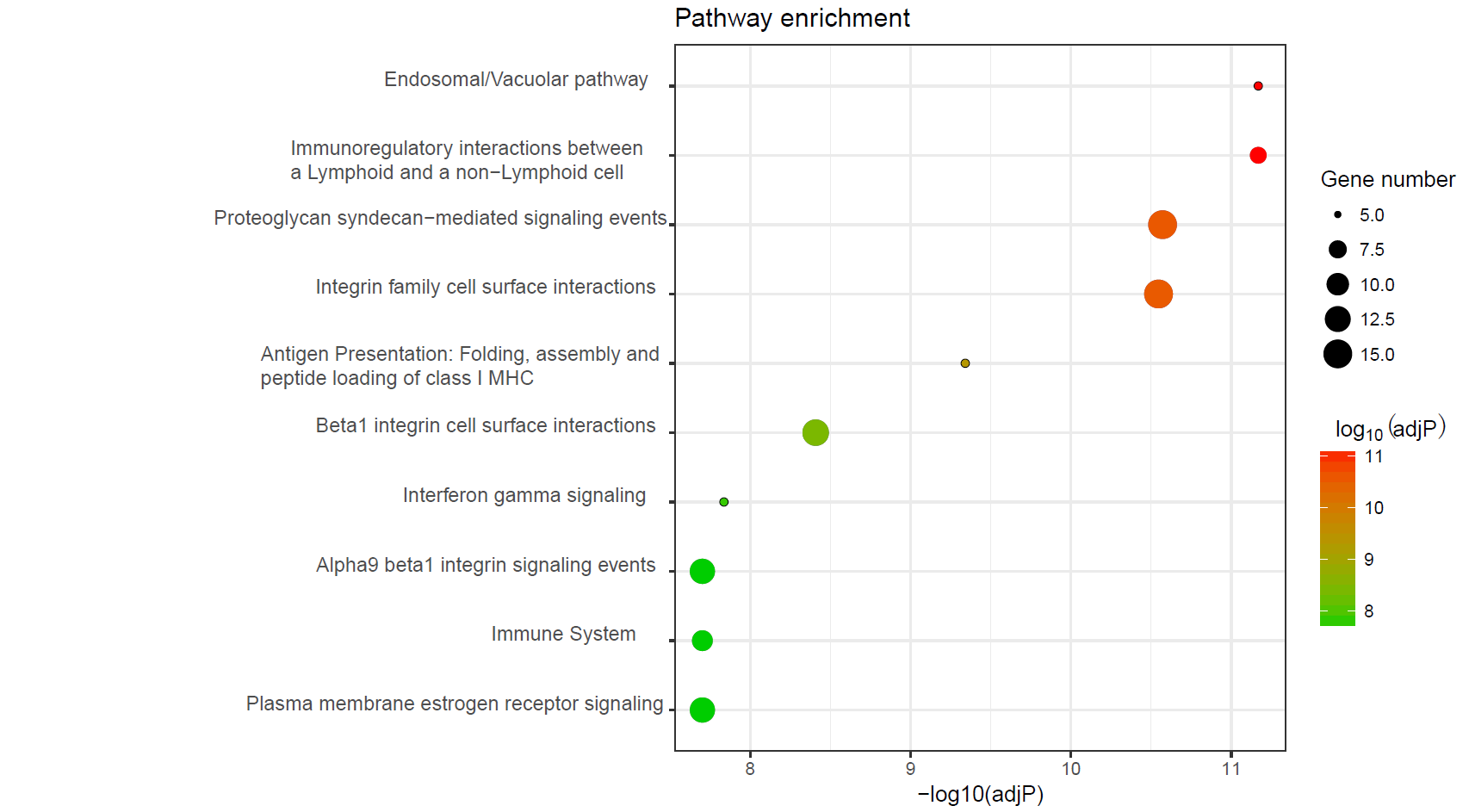


**Supplementary Figure 4.** Enriched pathways for ligands highly expressed in macrophages and receptors highly expressed in stem-like cells (Pathway commons).


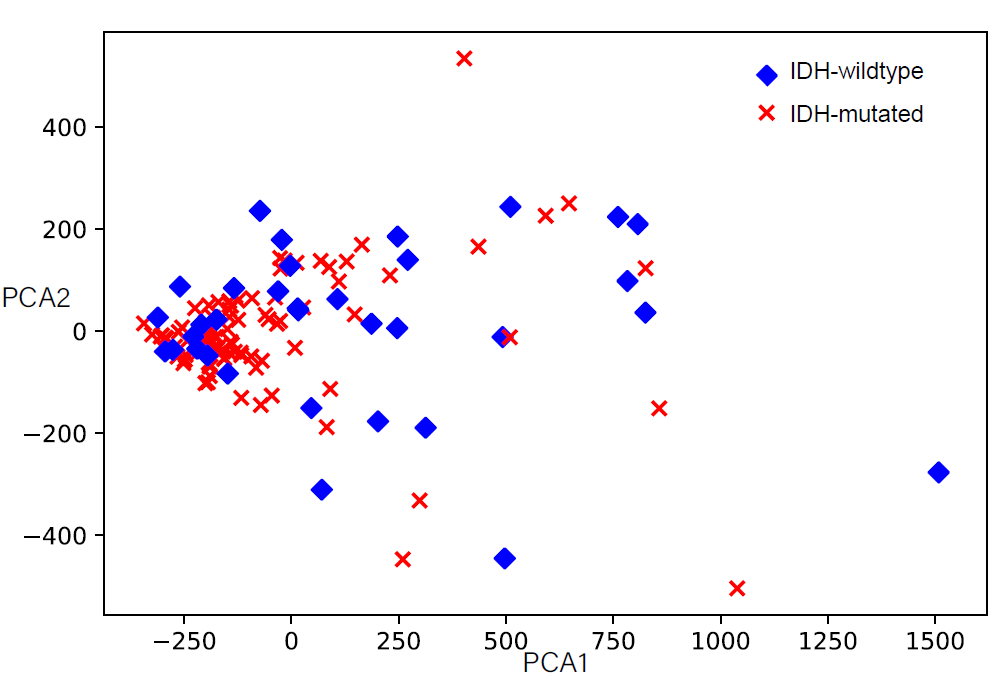


**Supplementary Figure 5.** PCA of IDH-mutated and IDH-wildtype gliomas based on the genes of ligand-receptor pairs.

**

**Supplementary Figure 6.** Performance of model built by randomly selected genes.

**Table 1.** 16 autocrine ligand-receptor pairs with significant Spearman’s correlation coefficients higher than 0.4. the first time they are cited.

| **Ligand** | **Receptor** | **Spearman’s correlation coefficients** | **p-value** | **Adjusted-p-value** |
| --- | --- | --- | --- | --- |
| BMP2 | ACVR2B | 0.5484285 | 1.83018E-41 | 3.25E-41 |
| COL1A1 | ITGA11 | 0.5565861 | 6.6373E-43 | 1.33E-42 |
| COL1A2 | ITGA11 | 0.5741402 | 3.83312E-46 | 1.02E-45 |
| DLL1 | NOTCH1 | 0.6052697 | 2.16468E-52 | 1.15E-51 |
| EFNA3 | EPHA7 | 0.4022327 | 2.70858E-21 | 2.89E-21 |
| GDNF | GFRA1 | 0.4012404 | 3.45835E-21 | 3.46E-21 |
| NCAM1 | GFRA1 | 0.445945 | 2.44658E-26 | 3.26E-26 |
| NLGN1 | NRXN1 | 0.4149346 | 1.10234E-22 | 1.36E-22 |
| NLGN2 | NRXN2 | 0.5934062 | 6.26581E-50 | 2.01E-49 |
| NLGN3 | NRXN2 | 0.6698561 | 8.0013E-68 | 6.40E-67 |
| NXPH1 | NRXN1 | 0.557782 | 4.05002E-43 | 9.26E-43 |
| NXPH1 | NRXN2 | 0.4117269 | 2.50662E-22 | 2.86E-22 |
| RPH3A | NRXN1 | 0.4764927 | 2.5274E-30 | 3.68E-30 |
| RTN4 | CNTNAP1 | 0.6893695 | 2.76075E-73 | 4.42E-72 |
| RTN4 | RTN4R | 0.5201512 | 9.1462E-37 | 1.46E-36 |
| SLIT1 | ROBO1 | 0.6033695 | 5.45302E-52 | 2.18E-51 |

**Table 2.** 90 genes associated with stemness in glioma

| SOX4, DCX, IGFBPL1, SOX11, TCF4, NREP, RND3, CCND2, MIAT, CAMK2N1, STMN4, STMN1, MYT1L, HN1, RNF122, PROX1, KLHDC8A, ELAVL4, NMNAT2, TUBB, ROBO1, NELL2, MLLT11, CELF4, POU3F2, H3F3B, ENC1, GNG2, ACOT7, AKT3, ARL4C, FNBP1L, VOPP1, TOX3, TUBB3, SCG2, TMSB15A, TFDP2, TMSB4X, CDC42, STMN2, KCTD13, RPH3A, KIF5C, NFIX, CALM1, TNPO2, BOC, KLHL13, PGAP1, RBFOX2, TMSB10, DYNLT1, TMSB15B, TCEAL7, PTS, BICD1, UCHL1, COMMD3, MCM7, AMZ2, PDRG1, DDAH2, KLC1, PCSK2, OAZ1, TIMM17A, YWHAG, CBX1, SMS, DGUOK, SNRPG, CDK6, GOLT1B, DUSP10, ATP5J, DYNLRB1, TCP1, GADD45G, SEC31A, CNOT7, DDX39A, SRGAP2, MAST2, PGK1, CELF3, ZFAS1, ENO2, SNRPB, DRG1 |
| --- |
